# Methyl Caffeate Isolated from the Flowers of *Prunus persica* (L.) Batsch Enhances Glucose-Stimulated Insulin Secretion

**DOI:** 10.3390/biom11020279

**Published:** 2021-02-14

**Authors:** Dahae Lee, Yutong Qi, Ranhee Kim, Jungbin Song, Hocheol Kim, Hyun Young Kim, Dae Sik Jang, Ki Sung Kang

**Affiliations:** 1 College of Korean Medicine, Gachon University, Seongnam 13120, Korea; pjsldh@gachon.ac.kr; 2 Department of Life and Nanopharmaceutical Sciences, Graduate School, Kyung Hee University, Seoul 02447, Korea; qiyutong9675@gmail.com (Y.Q.); rhee0423@khu.ac.kr (R.K.); 3 Department of Herbal Pharmacology, College of Korean Medicine, Kyung Hee University, Seoul 02447, Korea; jbsong@khu.ac.kr (J.S.); hckim@khu.ac.kr (H.K.); 4 Department of Food Science, Gyeongnam National University of Science and Technology, Jinju 52725, Korea; hykim@gntech.ac.kr

**Keywords:** *Prunus persica* (L.) Batsch, methyl caffeate, insulin, PI3K, AKT, PPARγ, PDX-1

## Abstract

Phenolic compounds from natural products are considered effective enhancers of insulin secretion to prevent and treat type 2 diabetes (T2DM). The flowers of *Prunus persica* (L.) Batsch also contain many phenolic compounds. In this study, the extract of flowers of *P. persica* (PRPE) exhibited an insulin secretion effect in a glucose-stimulated insulin secretion (GSIS) assay, which led us to isolate and identify the bioactive compound(s) responsible for these effects. Compounds isolated from PRPE were screened for their efficacy in INS-1 rat pancreatic β-cells. Among them, caffeic acid (5), methyl caffeate (6), ferulic acid (7), chlorogenic acid (8), naringenin (11), nicotiflorin (12), and astragalin (13) isolated from PRPE increased GSIS without inducing cytotoxicity. Interestingly, the GSIS effect of methyl caffeate (6) as a phenolic compound was similar to gliclazide, an antidiabetic sulfonylurea drug. Western blot assay showed that methyl caffeate (6) enhanced the related signaling proteins of the activated pancreatic and duodenal homeobox-1 (PDX-1) and peroxisome proliferator-activated receptor-γ (PPAR-γ), but also the phosphorylation of the total insulin receptor substrate-2 (IRS-2), phosphatidylinositol 3-kinase (PI3K), and Akt, which influence β-cell function and insulin secretion. This study provides evidence that methyl caffeate (6) isolated from PRPE may aid in the management of T2DM.

## 1. Introduction

The incidence of type 2 diabetes (T2DM), about 90% of all diabetes cases, is constantly increasing and becoming a social problem as it is a life-threatening disease accompanied by complications such as kidney damage and slow-healing wounds [1]. T2DM is a result of pancreatic islet β-cell dysfunction and insulin resistance, which can lead to a deficiency in insulin secretion in response to glucose [2]. Pancreatic islet β-cells are characterized by basal secretion when unstimulated, in conditions of low glucose concentration, and in increased secretion when stimulated by high glucose concentration. These cells play an important role in blood glucose control by secreting insulin and accepting insulin regulation [3]. Thus, identifying compounds that act like insulin, enhance pancreatic islet β-cell regeneration, or increase insulin secretion can help to develop strategies to prevent and treat T2DM. Recently, natural products have gained attention as important resources as potential anti-T2DM bioactive compounds.

Natural ingredients have been isolated from herbal medicine with anti-T2DM properties, and their possible mechanisms have been elucidated [4,5]. Polypeptide-p isolated from *Momordica charantia*, a bioactive natural ingredient from herbal medicine, has been reported to act as insulin [6], and epicatechin isolated from *Pterocarpus marsupium* induces pancreatic islet β-cell regeneration [7]. S-methylcysteine sulfoxide isolated from *Allium cepa* [8], Momordicoside U isolated from *Momordica charantia* [9], stigmasterol-3-*O*-β-d-glucoside isolated from the fruit of *Akebia quinata* [10], formononetin isolated from *Astragalus membranaceus* [11], and hypoxylonol F isolated from *Annulohypoxylon annulatum* [12] have been reported as bioactive compounds that increase insulin secretion from pancreatic islet β-cells. These bioactive natural ingredients that increase insulin secretion also enhance the expression of proteins related to the regulation of pancreatic β-cell survival and function, such as peroxisome proliferator-activated receptor-γ (PPARγ), insulin receptor substrate-2 (IRS-2), phosphatidylinositol 3-kinase (PI3K), Akt, and pancreatic and duodenal homeobox-1 (PDX-1).

The flower of *Prunus persica* (L.) Batsch has long been used in Chinese herbal medicine to treat skin disorders [13]. In previous studies based on this, the flower of *P. persica* has shown a protective effect from ultraviolet radiation and an inhibitory effect on melanogenesis on skin [13,14,15]. However, antidiabetic effects have yet to be demonstrated for its extract and its isolated compounds. The flowers of *P. persica* contain phenolic compounds, which in many plants have been reported to possess antidiabetic effects. Caffeic acid [16,17], naringenin [17], ferulic acid [18], astragalin [19], and chlorogenic acid have been reported to increase insulin secretion [20]. It has been reported that methyl caffeate induces pancreatic islet β-cell regeneration [21].

These previous studies propose that extracts of *P. persica* (PRPE) and phenolic compounds isolated from it are likely to be effective in increasing insulin secretion from pancreatic β cells.

In the current study, we found that PRPE showed an insulin secretion effect in a screening test, which led us to isolate and identify the bioactive compound(s) responsible for these effects. Next, a glucose-stimulated insulin secretion (GSIS) assay was conducted on INS-1 rat pancreatic β-cells to determine which of the isolated compounds contributed to the insulin secretion effect. After the preliminary screening, we focused on methyl caffeate, which showed similar effects to an antidiabetic sulfonylurea drug (gliclazide) and evaluated how it affects protein expression, including that of PPARγ, IRS-2, PI3K, Akt, and PDX-1.

## 2. Materials and Methods

### 2.1. General Experimental Procedures

NMR spectra were obtained using a 500 MHz (JEOL, Tokyo, Japan). Optical rotations were evaluated on a Jasco P-2000 polarimeter (JASCO, Tokyo, Japan) using a 10-cm microcell. Thin layer chromatography (TLC) analyses were performed on silica gel 60 F_254S_ (Merck, Kenilworth, NJ, USA) and RP-18 F_254S_ (Merck) plates. The compounds were visualized by dipping the plates into 20% (*v*/*v*) H_2_SO_4_ reagent (Aldrich, St. Louis, MI, USA) and then heated at 123 °C for 10–15 min. Diaion HP-20 (Mitsubishi, Tokyo, Japan) and Sephadex LH-20 (Amersham Pharmacia Biotech, Amersham, UK) were used for column chromatography. Flash chromatography was performed using a flash purification system (Combi Flash Rf; Teledyne Isco, Lincoln, NE, USA) with pre-packed cartridges, Redi Sep-C18 (26 and 43 g; Teledyne Isco). All solvents used for the chromatographic separations were distilled before use.

### 2.2. Plant Material

The dried flowers of *P. persica* Batsch (Rosaceae) were purchased from a Chinese herbal market (Xian, Shaanxi province, China) and authenticated by one of the authors H.K. A voucher specimen (PRPE-2018) of the raw material was deposited in the Laboratory of Natural Product Medicine, College of Pharmacy, Kyung Hee University, Seoul, Korea.

### 2.3. Extraction and Isolation

The dried flowers (100.0 g) of *P. persica* were extracted twice with distilled water at 100 °C for 2 h, and the solvent was evaporated in vacuo at 45 °C to obtain the hot water extract (PRPE, 23.5 g), which was then fractionated using Diaion HP-20 column chromatography (CC) (*ϕ* 4.6 × 32.3 cm, acetone-H_2_O = 40:60 to 60:40 *v*/*v*) to obtain eight fractions (F1–F8).

Fraction F1 (21.1 g) was separated by Sephadex LH-20 CC (*ϕ* 4.6 × 32.0 cm), with acetone-H_2_O (0:100 to 40:60 *v*/*v*), to obtain 10 subfractions (F1-1–F1-10). Subfraction F1-4 was fractionated using Sephadex LH-20 CC (*ϕ* 3.7 × 49.1 cm, 40% acetone) into compound **8** (150.7 mg) and 13 subfractions (F1-4-1–F1-4-13). Subfractions F1-4-5 was subjected to flash CC with Redi Sep-C18 cartridge (43 g, MeOH-H_2_O = 0:100 to 40:60 *v*/*v*) and produced compounds **1** (459.8 mg) and **9** (22.7 mg). Compound **2** (19.6 mg) was isolated by flash CC using a Redi Sep-C18 cartridge (26 g, acetonitrile-H_2_O = 0:100 to 30:70 *v*/*v*) from subfraction F1-4-4. Subfraction F1-4-8 was fractionated using a Redi Sep-C18 cartridge (43 g, acetonitrile-H_2_O = 0:100 to 40:60 *v*/*v*) to produce compounds **3** (99.3 mg) and **4** (125.6 mg). Compounds **5** (22.9 mg) and **6** (9.0 mg) were separated by Sephadex LH-20 CC (*ϕ* 3.9 × 89.0 cm, 40% acetone) from subfraction F1-3. Subfraction F1-2 was fractionated further using Sephadex LH-20 CC (*ϕ* 3.7 × 49.1 cm, 40% acetone) and was divided into five subfractions (F1-2-1–F1-2-5). Compound **15** (10.1 mg) was purified using a Redi Sep-C18 cartridge (43 g, MeOH-H_2_O = 0:100 to 20:80 *v*/*v*) from subfraction F1-2-5.

Fraction F2 (567.9 mg) was separated into 11 subfractions (F2-1–F2-11) using Sephadex LH-20 CC (*ϕ* 2.5 × 61.8 cm, MeOH-H2O = 80:20. Subfraction F2-5 was subjected to flash CC with Redi Sep-C18 cartridge (26 g, MeOH-H2O = 35:65 to 45:55 *v*/*v*) and generated compounds 7 (8.3 mg) and 10 (16.7 mg). Subfraction F2-5 was separated using a flash chromatographic system with a Redi Sep-C18 cartridge (26 g, MeOH-H2O = 35:65 to 45:55 *v*/*v*) to give compound 12 (4.3 mg). Compound 13 (13.3 mg) was purified from subfraction F2-8 using a Redi Sep-C18 column (26 g, MeOH-H2O = 0:0 to 45:55 *v*/*v*). Subfraction F2-9 was chromatographed on a flash chromatographic system with a Redi Sep-C18 cartridge (26 g, MeOH-H2O = 35:65 to 45:55 *v*/*v*) to produce compound 14 (20.9 mg).

Fraction F6 (112.3 mg) was further fractionated using a Sephadex LH-20 column (ϕ 2.5× 61.0 cm, 80% MeOH) to isolate compound 11 (15.1 mg).

### 2.4. Cell Culture

INS-1 cells from an immortalized rat insulinoma pancreatic β-cell line were purchased from Biohermes (Shanghai, China). INS-1 cells were cultured in a monolayer format in a Roswell Park Memorial Institute (RPMI) 1640 medium (Cellgro, Manassas, VA, USA) supplemented with 1% penicillin/streptomycin (Invitrogen Co., Grand Island, NY, USA), 10% fetal bovine serum (FBS), 2 mM L-glutamine, 0.05 mM 2-mercaptoethanol, 11 mM D-glucose, 10 mM 4-(2-hydroxyethyl)-1-piperazineethanesulfonic acid (HEPES) and 1 mM sodium pyruvate in a humidified atmosphere at 37 °C containing 5% CO_2_.

### 2.5. Cell Viability

The Ez-Cytox cell viability assay kit (Daeil Lab Service Co., Seoul, Korea) was used to determine the cytotoxicity concentration of PRPE and compounds **1**–**15** on INS-1 cells before their use in the glucose-stimulated insulin secretion (GSIS) assay. Compounds **1**–**15** were dissolved in dimethyl sulfoxide (DMSO) at a concentration of 100 mM. This was used as a stock solution, further diluted 100 times or more in a cell culture medium, and 0.5% DMSO was used as a solvent control in the untreated cells. INS-1 cells were seeded at a density of 10,000 cells per well in 96-well plates for 24 h. Afterwards, INS-1 cells were treated with different concentrations of PRPE and compounds **1**–**15** for 24 h. Cell viability was determined by incubating INS-1 cells in 10% (*v*/*v*) Ez-Cytox reagent for 2 h. Thereafter, the optical density (OD) at 450 nm was measured using a microplate reader (PowerWave XS, Bio-Tek Instruments, Winooski, VT, USA).

### 2.6. Glucose-Stimulated Insulin Secretion Assay

A rat insulin ELISA kit (Gentaur, Shibayagi Co. Ltd., Gunma, Shibukaw, Japan) was used to determine the insulin secretory function of PRPE and compounds **1**–**15** on INS-1 cells. INS-1 cells were seeded at a density of 400,000 cells per well in 12-well plates for 24 h. Afterwards, INS-1 cells were first incubated in a Krebs–Ringer bicarbonate buffer (KRB, pH 7.4) and starved for 1 h. The KRB was then removed and preincubated with PRPE, compounds **1**–**15**, or gliclazide for 30 min, then stimulated for 1 h with KBR with low glucose (3.3 mM) or high glucose (16.7 mM), respectively, as an INS-1 cell stimulant. The KRB was collected, and the insulin concentration in KRB was measured using a rat insulin ELISA kit in accordance with the manufacturer’s instructions. The optical density at 450 nm was measured using a microplate reader (PowerWave XS, Bio-Tek Instruments, Winooski, VT, USA). The glucose stimulation index (GSI) was calculated as follows: GSI = insulin level in high glucose (16.7 mM)/insulin level in low glucose (3.3 mM).

### 2.7. Western Blot Analysis

Western blot analysis was performed to determine the effect of methyl caffeate (**6**) on protein expression related to pancreatic β-cell metabolism. INS-1 cells were seeded at a density of 800,000 cells/well in 6-well plates for 24 h, then treated with different concentrations of methyl caffeate (**6**) for 24 h and finally collected. Total protein was extracted in cold RIPA buffer (Cell Signaling, Danvers, MA, USA) containing 1 mM phenylmethylsulfonyl fluoride. Protein samples were separated on a 10% sodium dodecyl sulfate polyacrylamide gel. The separated proteins were transferred onto polyvinylidene difluoride membranes. The membrane was incubated with primary antibodies against PDX-1, Phospho-IRS2 (P-IRS2) (Ser731), IRS-2, Phospho-PI3K (P-PI3K), PI3K, phospho-Akt (P-Akt) (Ser473), Akt, and glyceraldehyde 3-phosphate dehydrogenase (GAPDH) overnight at 4 °C, then incubated with horseradish peroxidase (HRP)-conjugated anti-rabbit secondary antibodies at room temperature for 1 h. The target protein band on the membrane was developed using ECL Plus Western blotting detection reagents (GE Healthcare, Little Chalfont, UK), and the immunoreactive bands were visualized using a chemiluminescence system (FUSION Solo, PEQLAB Biotechnologie GmbH, Erlangen, Germany).

### 2.8. Statistical Analysis

All experiments were performed in triplicates. All analyses were performed using SPSS Statistics ver. 19.0 (SPSS Inc., Chicago, IL, USA). Nonparametric comparisons of samples were conducted using the Kruskal–Wallis test to analyze the results. A value of *p* < 0.05 was considered to be statistically significant.

## 3. Results

### 3.1. Identification of Compounds **1**–**15**

**Figure 1:**
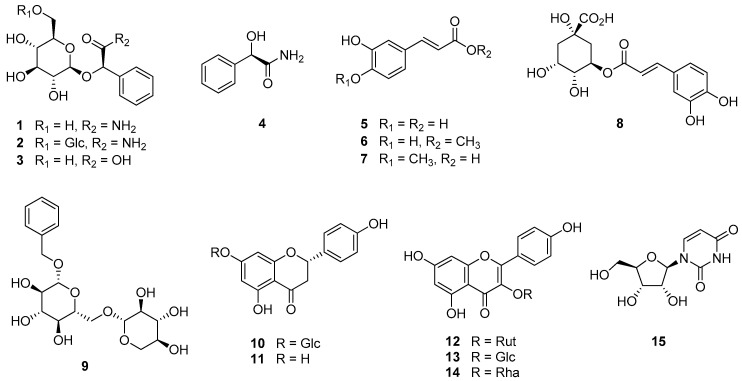
Chemical structures of compounds **1**–**15** isolated from the flowers of *P. persica*.

The chemical structures of the isolated compounds were identified by analysis of spectroscopic data (^1^H, ^13^C, and 2D NMR) measurements and a comparison with reported data (Table 1 and Figure 1).

### 3.2. Effects of PRPE and Compounds **1**–**15** on Glucose-Stimulated Insulin Secretion

**Figure 2:**
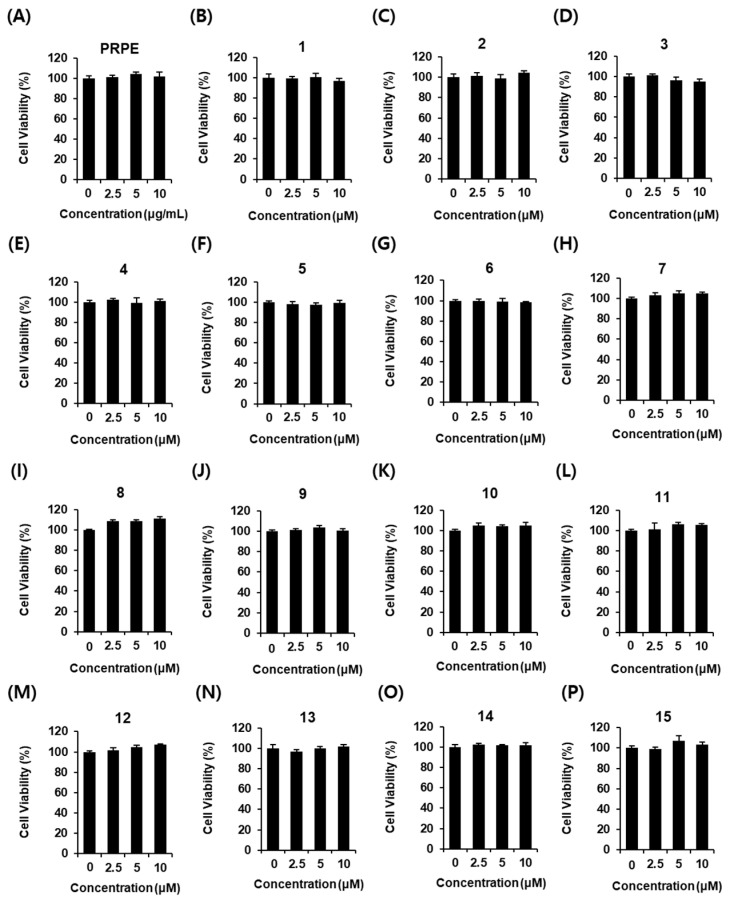
Effect of *P. persica* (PRPE) and compounds **1**–**15** on the viability of INS-1 cells. Effect of (**A**) PRPE, (**B**–**P**) compounds **1**–**15** compared with the control (0 μg/mL or 0 μM) on the viability of INS-1 cells for 24 h by 3-[4,5-dimethylthiazol-2-yl]-2,5 diphenyl tetrazolium bromide (MTT) assay (*n* = 3 independent experiments, *p* > 0.05, Kruskal–Wallis nonparametric test). Data represent the mean ± SEM.

PRPE and compounds **1**–**15** were tested for their effect on cell viability to select the non-toxic concentration to be used in the glucose-stimulated insulin secretion (GSIS) assay and did not show any toxicity at any concentration (Figure 2).

**Figure 3:**
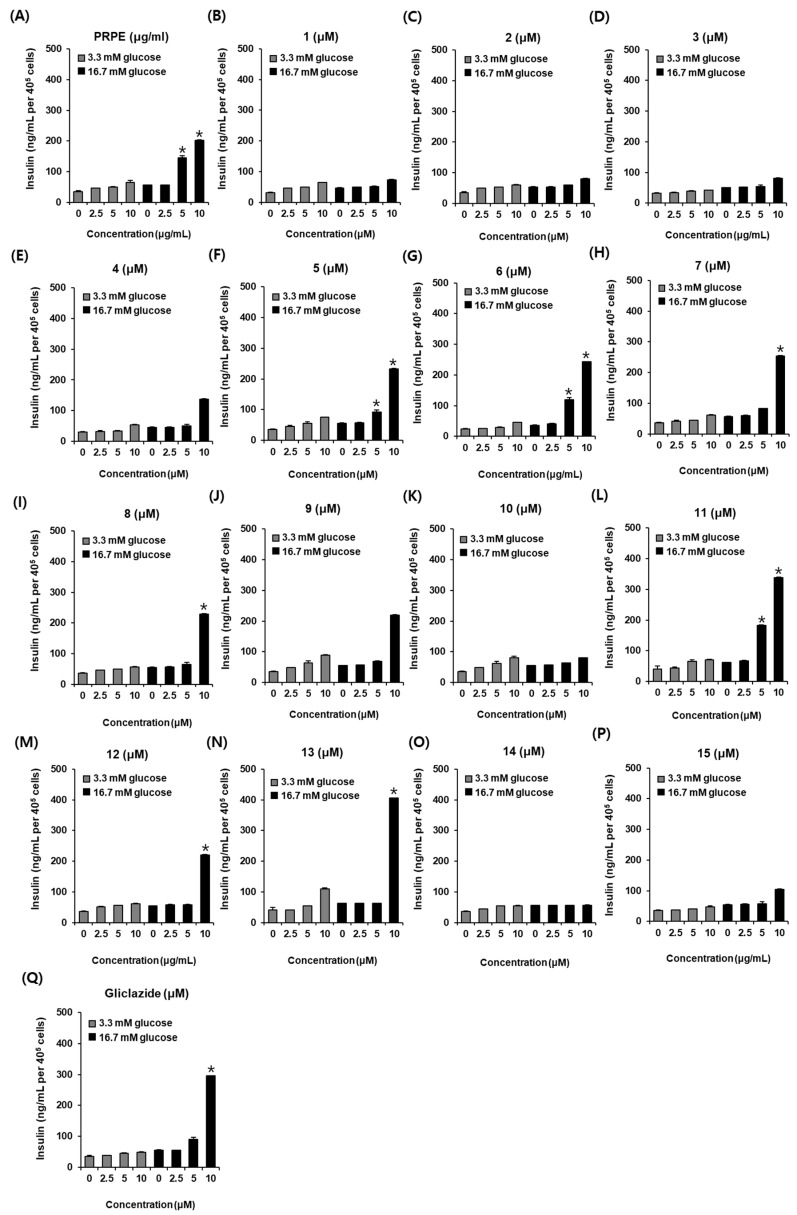
Effect of PRPE and compounds **1**–**15** on glucose-stimulated insulin secretion (GSIS, ng/mL per 400,000 cells) in INS-1 cells. Effect of (**A**) PRPE, (**B**–**P**) compounds **1**–**15**, and (**Q**) gliclazide (positive control) compared with the control (0 μg/mL or 0 μM) on GSIS (ng/mL per 400,000 cells) in INS-1 cells for 1 h by insulin secretion assay (*n* = 3 independent experiments, * *p* > 0.05, Kruskal–Wallis nonparametric test). Data represent the mean ± SEM.

**Figure 4:**
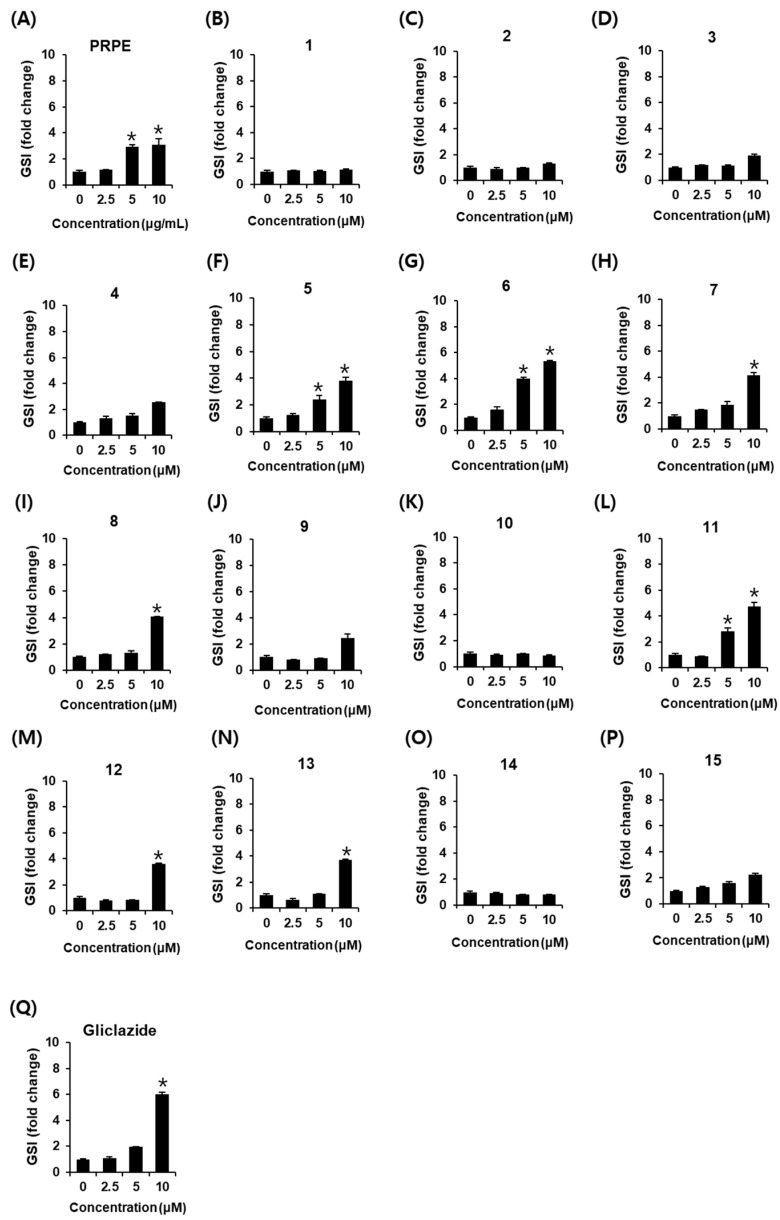
Effect of PRPE and compounds **1**–**15** on glucose-stimulated insulin secretion (GSIS) expressed as the glucose stimulation index (GSI) in INS-1 cells. Effect of (**A**) PRPE, (**B**–**P**) compounds **1**–**15**, and (**Q**) gliclazide (positive control) compared with the control (0 μg/mL or 0 μM) on GSIS expressed as the GSI in INS-1 cells for 1 h by insulin secretion assay. GSI = insulin level in high glucose (16.7 mM)/insulin level in low glucose (3.3 mM). (*n* = 3 independent experiments, * *p* > 0.05, Kruskal–Wallis nonparametric test). Data represent the mean ± SEM.

As shown in Figure 3, PRPE, compounds **5**–**8**, and compounds **11**–**13** increased GSIS (ng/mL per 400,000 cells). GSIS was expressed as the GSI. Fold change was set at 1 for control cells. As shown in Figure 4A, PRPE increased the GSI at concentrations of 5 and 10 μg/mL. At that concentration, the values of GSI were 2.94 ± 0.13 and 3.11 ± 0.43, respectively. This result indicated that PRPE contains compounds with GSIS activity. GSI values were: 2.39 ± 0.32 and 3.82 ± 0.23 for caffeic acid (**5**) at concentrations of 5 and 10 μM, respectively (Figure 4F); 4.01 ± 0.08 and 5.34 ± 0.06 for methyl caffeate (**6**) at a concentration of 5 and 10 μM, respectively (Figure 4G); 4.16 ± 0.18 for ferulic acid (**7**) at a concentration of 10 μM (Figure 4H); 4.05 ± 0.04 for chlorogenic acid (**8**) at a concentration of 10 μM (Figure 4I); 2.81 ± 0.29 and 4.75 ± 0.29 for naringenin (**11**) at concentrations of 5 and 10 μM, respectively (Figure 4L); 3.59 ± 0.07 for nicotiflorin (**12**) at a concentration of 10 μM (Figure 4M); and 3.71 ± 0.02 for astragalin (**13**) at a concentration of 10 μM (Figure 4N). Among these compounds, the GSI of methyl caffeate (**6**) was the highest, and its GSIS activity was similar to that of gliclazide (6.01 ± 0.16 at a concentration of 10 μM, Figure 4Q): methyl caffeate (**6**) was selected as the subject of further mechanistic studies.

### 3.3. Effect of Methyl Caffeate (**6**) on the Protein Expression of PPARγ, P-IRS-2, IRS-2, P-PI3K, PI3K, P-Akt (Ser473), Akt, and PDX-1

**Figure 5:**
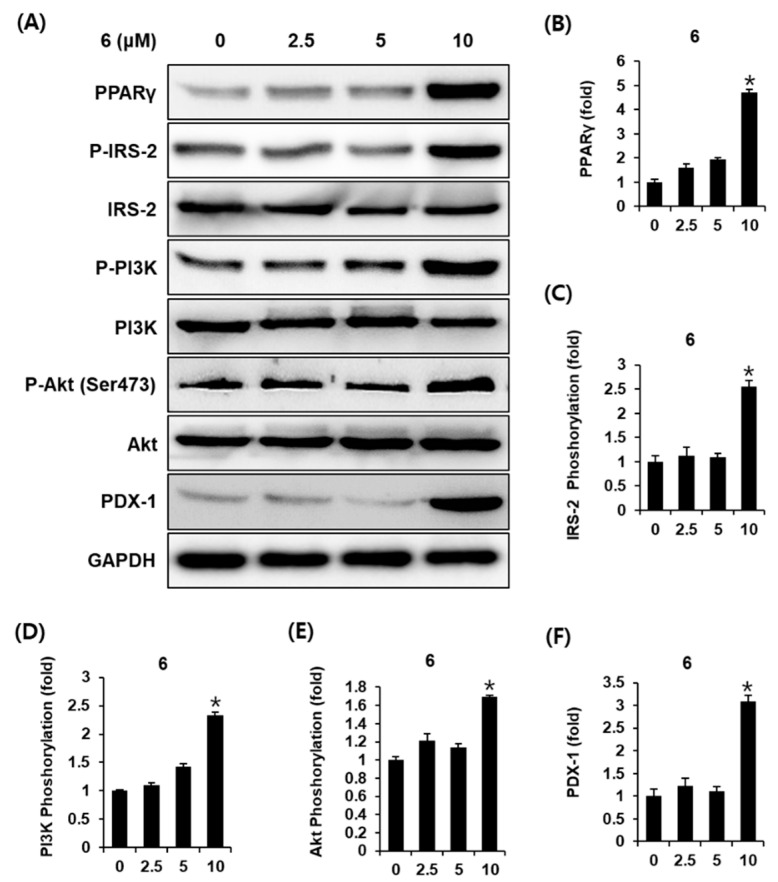
Effect of methyl caffeate (**6**) on the protein expression levels of peroxisome proliferator activated receptor γ (PPARγ), phospho-insulin receptor substrate-2 (P-IRS-2) (Ser731), IRS-2, phospho-phosphatidylinositol 3-kinase (P-PI3K), PI3K, phospho-Akt (P-Akt) (Ser473), and Akt, and pancreatic and duodenal homeobox-1 (PDX-1) in INS-1 cells. (**A**) Protein expression levels of P-IRS-2 (Ser731), IRS-2, P-PI3K, PI3K, P-Akt (Ser473), Akt, pancreatic and duodenal homeobox-1 (PDX-1), and glyceraldehyde 3-phosphate dehydrogenase (GAPDH) in INS-1 cells treated or untreated with 2.5 μM, 5 μM, and 10 μM methyl caffeate (**6**) for 24 h. (**B**–**F**) The bar graph presents the densitometric quantification of Western blot bands (*n* = 3 independent experiments, * *p* > 0.05, Kruskal–Wallis nonparametric test). Data represent the mean ± SEM.

To explore the underlying influence of methyl caffeate (**6**) on glucose-stimulated insulin secretion in INS-1 cells, the expression of proteins related to pancreatic β-cell metabolism was analyzed. As shown in Figure 5A, 10 μM methyl caffeate (**6**) increased the relative abundances of peroxisome proliferator activated receptor γ (PPARγ), phospho-insulin receptor substrate-2 (P-IRS-2) (Ser731), phospho-phosphatidylinositol 3-kinase (P-PI3K), phospho-Akt (P-Akt) (Ser473), and pancreatic and duodenal homeobox-1 (PDX-1) proteins. The bar graphs illustrate the ratio of PPARγ, P-IRS-2, P-PI3K, P-Akt (Ser473), and PDX-1 expression normalized to their corresponding GAPDH expression (Figure 5B–F). These results suggest that the effect of methyl caffeate (**6**) enhances the expression of proteins related to pancreatic β-cell metabolism.

## 4. Discussion

The results of this study indicated that PRPE increased GSIS, which led us to isolate and identify the bioactive compound(s) responsible for these effects. Among the isolated compounds, caffeic acid (**5**), methyl caffeate (**6**), ferulic acid (**7**), chlorogenic acid (**8**), naringenin (**11**), nicotiflorin (**12**), and astragalin (**13**) increased GSIS. Our results are in agreement with previous studies, which showed that caffeic acid (**5**) [16,17], ferulic acid (**7**) [18], chlorogenic acid (**8**), naringenin (**11**) [17], and astragalin (**13**) [19] have been reported to increase insulin secretion [20]. Whereas each compound was previously isolated from a different plant, in our study, PRPE with GSIS effect contained all these compounds, with the addition of methyl caffeate (**6**), chlorogenic acid (**8**), and nicotiflorin (**12**), for which no GSIS effect was reported.

Interestingly, methyl caffeate (**6**) has a better effect than its free acid form, caffeic acid (**5**). Several studies on the biological activities of methyl caffeate (**6**) have reported its neuroprotective [36], anticancer [37], antioxidant [38], and anti-inflammatory effects [39] as well as antimicrobial and antimycobacterial activities [40]. Additionally, the induction of pancreatic islet β-cell regeneration by methyl caffeate (**6**) has also become clear [21], as its oral administration improved glucose tolerance in rats. It has been speculated, but still not proved, that this effect is mediated through insulin secretion from pancreatic islet β-cells. In this study, the GSIS effect of methyl caffeate (**6**) was similar to that of gliclazide, an antidiabetic sulfonylurea drug. Based on the GSIS assay, we have shown the possible GSIS activity of methyl caffeate (**6**) GSIS by modulating cell secretory capacity. These findings distinguish our study from previously reported antidiabetic effects of methyl caffeate (**6**).

In this study, Western blot analysis was performed to determine the effect of methyl caffeate (**6**) on protein expression related to pancreatic β-cell metabolism, such as PPARγ, IRS-2, PI3K, Akt, and PDX-1. According to published studies, synthetic ligands for PPAR-γ influence GSIS, as PPAR-γ activation upregulates the GPR40 receptor in INS-1 cells [41,42]. In addition, the gene expression of pancreatic β-cells is upregulated through PPAR-γ activation [41]. PPAR-γ agonists have been shown to have a direct effect on improving pancreatic β-cell survival and function [43]. An increase in insulin secretion induced by rosiglitazone, a thiazolidinedione antihyperglycemic agent, is mediated through the PPAR-γ pathway in INS-1 cells [44]. Our study also showed a clear increase in the expression level of PPAR-γ in INS-1 cells treated with methyl caffeate (**6**).

IRS-2 closely regulates pancreatic β-cell growth and development [45]. Deletion of IRS-2 in mice leads to pancreatic β-cell mass [46]. Because β-cell mass grows into adulthood to meet increased insulin demand, adequate islet mass is essential for insulin secretion [47]. In addition, IRS-2 activates Akt in a PI3K-dependent manner. The PI3K/Akt pathway has been shown to regulate early-phase insulin secretion [48], β-cell mass, and function. The expression of Akt is also required for β-cell regeneration [49]. Recent evidence has demonstrated that rosiglitazone enhances GSIS in the pancreas of rats through PI3K activation [50]. A previous study reported that silibinin, a flavonoid, can improve β-cell functionality and increase GSIS through the upregulation of IRS-2 and PDX-1 [51]. Studies have further indicated that imeglimin derivatives promote GSIS, which is associated with an overexpressed IRS-2, while promoting Akt [52]. Our study also showed that methyl caffeate (**6**) significantly increased the expression of IRS-2, PI3K, and Akt in treated INS-1 cells.

Another important role of the IRS-2/PI3K/Akt pathway is the regulation of nuclear translocation of endogenous PDX-1. A previous study demonstrated that the key gene involved in β-cell functionality and insulin synthesis is PDX-1, a β-cell transcription factor. In IRS-2 knockout mice, transgenic expression of PDX-1 restores β-cell function, including insulin secretion [53]. In agreement with the positive effects of overexpressed PDX-1 on GSIS by treatment with resveratrol, a polyphenolic compound [54], the present study found that the protein expression of PDX-1 increased after treatment with methyl caffeate (**6**) in the same pattern as the protein expression of IRS-2, PI3K, and Akt. Therefore, the potentiation effect of methyl caffeate (**6**) on the GSIS might be explained by the upregulation of PPARγ and PDX-1 via the IRS-2 signaling pathways in INS-1 cells, which play an important role in β-cell function for insulin-secretory capacity to promote GSIS.

## 5. Conclusions

Our results showed that PRPE and caffeic acid (**5**), methyl caffeate (**6**), ferulic acid (**7**), chlorogenic acid (**8**), naringenin (**11**), nicotiflorin (**12**), and astragalin (**13**) isolated from PRPE enhanced GSIS in INS-1 cells. Interestingly, the GSIS effect of methyl caffeate (**6**) was similar to that of gliclazide, an antidiabetic sulfonylurea drug. INS-1 rat pancreatic β-cells treated with caffeic acid (**5**) also enhanced the induction of PPARγ and PDX-1 via the IRS-2 signaling pathways related to survival and function of pancreatic β-cells. Ultimately, these promising findings highlighted caffeic acid (**5**) isolated from PRPE as a possible drug candidate to treat T2DM after its promising results in vivo. Despite these findings, further investigations are needed to check whether methyl caffeate (**6**) can increase insulin gene expression or insulin content, and more studies are needed to prove the effect of methyl caffeate (**6**) in animal models for studies of T2DM.

## Figures and Tables

**Table 1:** Compounds **1**–**15** isolated from the flowers of *P. persica*.

Name	Amount Obtained (Yield %/Extract)	Name	Amount Obtained (Yield %/Extract)
Prunasin amide (**1**) [22]	459.8 mg (1.957%)	Benzyl α–L-xylpyranosyl-(1→6)-β-glucopyranoside (**9**) [23]	22.7 mg (0.097%)
Amygdalin amide (**2**) [24]	19.6 mg (0.083%)		
Prunasin acid (**3**) [22]	99.3 mg (0.423%)	Prunin (**10**) [25]	16.7 mg (0.071%)
Mandelamide (**4**) [24,26]	125.6 mg (0.534%)	Naringenin (**11**) [27]	15.1 mg (0.064%)
Caffeic acid (**5**) [28]	22.9 mg (0.097%)	Nicotiflorin (**12**) [29]	4.3 mg (0.018%)
Methyl caffeate (**6**) [30]	9.0 mg (0.038%)	Astragalin (**13**) [31]	17.6 mg (0.075%)
Ferulic acid (**7**) [32]	8.3 mg (0.035%)	Afzelin (**14**) [33]	20.9 mg (0.089%)
Chlorogenic acid (**8**) [34]	150.7 mg (0.641%)	Uridine (**15**) [35]	10.1 mg (0.043%)

## Data Availability

The data presented in this study are available on request from the corresponding author.
